# Maternal Factors Associated with Dietary Diversity Scores of Children aged 6–23 Months in Kwale County, Kenya

**DOI:** 10.24248/eahrj.v7i2.727

**Published:** 2023-11-30

**Authors:** Francesca Chepkirui, Justus Osero, Lilian Nyandieka, Mami Hitachi, Satoshi Kaneko, Norah Wekesa, Juma Changoma, Violet Wanjihia

**Affiliations:** a School of Public Health, Department of Community Health and Epidemiology Kenyatta University, Nairobi Kenya; b Centre for Public Health Research, Kenya Medical Research Institute (KEMRI), Nairobi, Kenya; c Department of Eco-epidemiology, Institute of Tropical Medicine (NEKKEN), Nagasaki University, Nagasaki, Japan; d Kenya Medical Research Institute (KEMRI) Graduate School, Nairobi, Kenya; e Nagasaki University, Kenya research Station, NUITM-KEMRI Project, Kwale Kenya

## Abstract

**Background:**

Dietary Diversity (DD) is an important component of Infant and Young Child Nutrition (IYCN). Globally, it is recommended that children aged 6–23 months be fed on diverse diets as a public health measure in curbing malnutrition. In Kenya, stunting rates among children below the age of five years is 26% and diversifying of diets is still sub-optimal. The study sought to assess maternal factors associated with dietary diversity scores among children aged 6–23 months in Kwale County, where stunting stands at 29%.

**Methods:**

A cross sectional study design was adopted and a random sample of 244 mothers with children aged 6–23 months, residing in locations under Health and Demographic System Surveillance (HDSS) program participated in the study.

**Results:**

Mothers aged 17–68 years were interviewed. Children's DD scores ranged from 0 to 5 with a mean of 2.2±0.9, only 8.2% of the children met the Minimum Dietary Diversity score (MDDs) of 4 food groups or more in a day. A bivariate regression analysis to determine factors associated with children's dietary diversity scores showed significant positive relationship with mother's educational level (r=0.186, P<.000, α=.01), household wealth index (r=0.163, P<.000, α=.01) and the child's age (r=0.396, P<.004, α=.01).

**Conclusion:**

The study concludes that mothers' level of education, households' wealth index and child's age are factors associated with dietary diversity scores of children. Higher dietary diverse scores were observed among older children in the study age categories, from wealthy families and under care of mothers with higher education levels. In the study area however, only 8.2% of children met the minimum dietary diversity score necessitating targeted nutrition education of mothers and implementation of economic development initiatives to boost availability and consumption of diverse diets. A consideration of child age specific interventions will also address nutritional needs and preferences at different stages.

## INTRODUCTION

Globally, malnutrition is a public health problem. In developing countries, malnutrition is associated with 41% annual mortalities in children aged 6–23 months.^1^ Global malnutrition rates indicates 22.9% stunting and 7.7% wasting with Africa rates at 31.2% stunting and 7.4% wasting.^2^ Many factors have been attributed to cause malnutrition; one amongst the major factors is lack of adequate nutrition.^3^ To solve the problem of inadequate nutrition, a global strategy for Infant and Young Child Feeding (IYCF) has been developed. ^4,8^ The strategy made several recommendations and one amongst the recommendation was provision of dietary diversity to children aged 6–23 months.^4^

Dietary diversity (DD) refers to consumption of a variety of foods within and across food groups. Consumption of a variety of foods has been related with a higher probability of an adequate intake of macro and micronutrients essential for bodily functions.^5^ DD is very critical for children at the age of 6–23 months because this period is often observed as a critical window in prevention of growth faltering and under nutrition.^6,7^

Kenya faces the challenge of ending all forms of malnutrition. One of the strategies the government of Kenya has adopted in effort to end this problem is the IYCF strategy.^8–10^ One indicator under this strategy is DD. Compliance with the recommended dietary diversity is still suboptimal with only 41% of children aged 6–23 months meeting the Minimum Dietary Diversity (MDD).^11^ Although this rate is an improvement from the previous rate of 32.3%, ^12^ more significant improvement ought to be achieved. Such significant improvements can be attained through in-depth understanding of the drivers of lack of adherence to recommended DD.^13,14^ Kwale County presented an ideal study site to understand these factors. Kwale County has reported persistent high levels of chronic malnutrition in children less than five years over the years.^11^

## METHODOLOGY

### Study Setting

The study was conducted in Matuga and Kinango sub-counties, Kwale County, Kenya. The county is divided into four sub-counties namely: Matuga, Msambweni, Kinango and Lungalunga. This research was conducted in purposively selected locations within an established Health and Demographic System Surveillance (HDSS) program. HDSS covers areas within three sub-counties which include Matuga, Kinango and LungaLunga, whose area is 384.9 km^2^ with 9000 households and about 50, 000 inhabitants as at October, 2013. The area lies between latitudes 4°17′S and 4°5′S and longitudes 39°15′E and 39°29′E.^15^ Matuga and Kinango sub-counties were purposively selected due to reported high rate of stunting in periodic HDSS surveys implemented in the area by the Kenya Medical Research Institute in collaboration with Nagasaki University. An area within Lunga Lunga sub-county was used to conduct pre-test. Although the HDSS program did not cover all four sub-counties in Kwale County, the up-to-date data platform provided a credible database for sampling of study participants. Additionally, it provided a base for practical community support programs that seek to improve health of the people in the area.

### Research Design

This study adopted a cross-sectional study design.

### Study Population

The target population included mothers with children aged 6–23 months, residents of Kwale County living within purposively selected locations in a HDSS program. Mothers who gave written and signed consent took part in the study. Mothers were targeted due to their direct involvement in infant's nutrition both in provision and feeding.

### Sampling Procedure

A simple random sample of participants was computer generated from the list of households in HDSS database, residents of purposively selected locations. Using Fischer's formula of sample size estimation, a sample of 240 was derived.^16^ A Random sample of 244 (mother-child pairs) participated in the study.

### Data Collection

Individual interviews were conducted using a semi-structured questionnaire, qualitative 24-Hour recall questionnaire and a dietary diversity questionnaire. The semi-structured questionnaire contained a mixture of open and closed-ended questions on sociodemographics, household economics, maternal nutrition knowledge and maternal perceptions on Infant and Young Child Nutrition (IYCN). The 24-Hour recall questionnaire was structured with open-ended questions to capture children's food consumption in the previous 24 hours. Dietary diversity questionnaire was filled based on data collected from 24 Hour recall.

### Validity

To ensure validity, study tools were reviewed by supervisors and subjected to a pre-test. The tools were also translated to Kiswahili for ease of interpretation during interviews. Additionally, prior to conducting interviews an informed consent was sought and a copy of signed consent documented.

### Reliability

Reliability was ensured through cross-checking, inspecting, and closely examining the pre-tested surveys to assess precision, appropriateness, thoroughness, integrity, and homogeneity of the queries. Before the commencement of the research, the questionnaires were modified as needed in response to the pre-test comments. In addition, the lead investigator gave study assistants full training and oversight, conducting daily debriefings.

### Data Analysis

Data analysis of individual questionnaires was conducted using IBM SPSS version 21.0 statistical software. Exploratory data techniques were employed at the initial stage of analysis to uncover the structure of data and identify outliers or unusually entered values. Proportions were used to summarize categorical variables. Chi-square tests, bivariate regression analysis and Spearman's bivariate correlation analysis were conducted to ascertain the variables associations where a *P* <0.05 was considered to be statistically significant.

### Ethical Considerations

Permission to carry out this study was obtained from Kenyatta University Ethical Review Committee (PKU/1043/11193) and a research permit obtained from NACOSTI (NACOSTI/P/20/4001). Research approval was also sought from Ministry of Health in Kwale County. Informed consent was obtained from each index child's mother prior to participation in the study.

## RESULTS

### Sociodemographic and Household Economic Characteristics of Respondents

As presented in Table 1, all respondents were females as per the study target population. Their ages ranged from 17 years to 68 years with a mean of 29.24 ± 9.6 years. By educational levels, almost one-in-three or 29.5% of the respondents did not have any formal education, 10.2% had lower primary education (class 1 – 4), 53.3% had upper primary education (class 5–8), 5.3% had secondary education while only 1.2% had post-secondary education. Most (54.1%) of the respondents were housewives. Most (63.4%) of the studied households had 5 – 10 members, almost one – third (29.2%) had less than 5 members and only a small percentage 7.4%) had more than 10 members in the household.

**TABLE 1:** Socio-demographic and Economic Characteristics of Respondents

Characteristics	Frequency	Percent
Age in years of respondents (n=242)		
<= 24	84	34.7
25 – 34	108	44.6
35 – 44	31	12.8
45 – 54	10	4.1
55 – 64	7	2.9
65+	2	0.8
Educational level of respondent (n=244)		
No formal education	72	29.5
Primary 1–4	25	10.2
Primary 5–8	130	53.3
Secondary 1–4	13	5.3
Tertiary - college/university	3	1.2
Madrassa	1	0.4
Occupation of the respondent (n=244)		
Employed	3	1.2
Casual worker	9	3.7
Housewife	132	54.1
Business	32	13.1
Farmer	54	22.1
Unemployed	13	5.3
Retiree	1	0.4
Household size (n=243)		
Less 5	71	29.2
5 – 10	154	63.4
More than 10	18	7.4
Main source of income for the household		
Formal employment	21	8.6
Small scale farming	82	33.6
Farming & selling produce	6	2.5
Small business	28	11.5
Petty trade	6	2.5
Casual labor	99	40.6
Relatives/remittances	1	0.4
Others	1	0.4
Approximate monthly expenditure		
Less 1,000	1	0.4
1,000 – 2,999	14	5.7
3,000 – 4,999	56	23
5,000 – 9,999	111	45.5
10,000 – 19,999	39	16
20,000 – 49,999	3	1.2
Not sure	20	8.2
Wealth index		
1st quintile (Poorest)	50	20.5
2nd quintile	23	9.4
3rd quintile	73	29.9
4th quintile	46	18.9
5th quintile (wealthiest)	52	21.3

An assessment of the respondents' economic characteristics is also presented in Table 1. Economic variables were operationalized as household income sources, income expenditure, and wealth quintiles. The wealth quintiles were computed using the principal component analysis using the household water and sanitation practices, ownership of assets and housing characteristics.

Casual labor (40.6%) and small-scale farming (33.6%) were the main sources of income for the households with 8.6% getting their income from formal employment. Majority of the households spent less than Kshs. 10,000 in the one month preceding the survey. The computed wealth index classified 20.5% in the first quintile (poorest) and 21.3% in the wealthiest quintile (5^th^ quintile).

### Children's Dietary Diversity Scores

Children's dietary diversity score was assessed based on seven food groups namely: grains, roots and tubers, legumes and nuts, dairy products, flesh foods, eggs, vitamin A rich fruits and vegetables, and other fruits and vegetables. The children's dietary diversity score ranged from 0 to 5 with a mean of 2.2±0.9. Nine children (3.7%) had a score of 0 having only breastfed or taken water, or tea without milk, all of which did not count in the food grouping, 20.5% of the children recorded a score of one (1) having only eaten from the grains, roots and tubers group, 39.8% ate foods from two food groups, 27.9% from three food groups, 7.8% from 4 food groups and only 0.4% from 5 food groups as shown on Table 2.

**TABLE 2:** Children's Dietary Diversity Scores and Maternal Nutritional Knowledge

Characteristics	Frequency (n=244)	Percent (%)
Number of food groups consumed (Dietary Diversity)		
0	9	3.7
1	50	20.5
2	97	39.8
3	68	27.9
4	19	7.8
5	1	0.4
Knowledge of food groups (Correct No. of Food Groups listed)		
0	216	88.5
1	6	2.5
2	7	2.9
3	15	6.1
Knowledge of food sources, Sources of energy foods (Carbohydrates)		
0	155	63.6
1	34	13.9
2	24	9.8
3	31	12.7
Sources of body building (Proteins)		
0	204	83.5
1	16	6.6
2	17	7.0
3	7	2.9
Sources of Protective foods (Vitamins)		
0	216	88.6
1	3	1.2
2	12	4.9
3	13	5.3
Mothers' level of knowledge (Cut-Offs)		
0–5 (Low Score)	222	91
6–9 (Medium Score)	17	7
10–12 (High Score)	15	2

Table 3 describes the different food group consumption by the children. Majority of the children (95.9%) consumed foods from the grains, roots and tubers group. Other than this group and the vitamin A rich fruits and vegetables food group (34.4%), other foods in the other food groups were consumed by less than one-third of the children.

**TABLE 3:** Food Groups Consumption by Children (Age – Group)

Food group	6 – 11 months	12 – 17 months	18 – 23 months	Overall	Χ - Significance
Grains, roots and tubers,	90.8	95.6	100	95.9	.009
Legumes and nuts,	10.5	20.6	46.0	27.9	.000
Dairy products	25.0	17.6	22.0	21.7	.563
Flesh foods	6.6	17.6	25.0	17.2	.006
Eggs	0.0	0.0	0.0	0.0	–
Vitamin A rich fruits and vegetables	14.5	41.2	45.0	34.4	.000
Other fruits and vegetables	21.1	17.6	21.0	21.1	.840

None of the children consumed eggs in the study sample. The distribution consumption prevalence of the foods from the food groups by the age of the children are also described in Table 3. Significantly fewer children in the age group 6–11 consumed foods from the Vitamin A rich fruits and vegetables group and the flesh foods groups than the other age groups. A significantly bigger proportion of the children consumed foods in the food groups of grains, roots and tubers and legumes and nuts than the other age groups.

Overall, only 8.2% of the children met the minimum dietary diversity score of 4 food groups or more. Significantly bigger proportion (14%) of the children in the age-group 18 – 23 months (χ= 7.594, df=2, *P*=.022) compared with age groups 6–11 months at 5.7% and 12–17 months at 4.4% met the minimum dietary diversity.

### Mothers' Nutrition Knowledge

Mothers' knowledge of food groups and knowledge of the food's rich in energy, body building foods and protective foods was assessed. Only 11.5% of the respondents could correctly name at least one food group. The protective food group which includes fruits and vegetables that are rich in minerals and vitamins were the most popular, mentioned by 10.7% followed by energy giving foods (7.4%) and body building foods at 7%.

Table 2 shows the distribution of the respondents by the correct number of food groups mentioned. Fifteen respondents (6.1%) were able to correctly cite 3 food groups, seven (2.9%) correctly mentioned 2 groups and six (2.9%) mentioned only 1 group. The rest (88.5%) did not know any of the food groups.

The respondents were further asked to name three examples of energy giving foods, body building foods and protective foods. Overall, 36.5% of the respondents correctly mentioned at least one example of food rich in energy with 13.9% listing one correct food, 9.8% listing two foods and 12.7% listing three foods correctly. Only 16.4% of the respondents correctly identified at least one example of body building foods. 6.6% of these identified correctly one example, 7% two examples and 2.9% three examples. On the other hand, 11.5% of the respondents gave correct examples of protective foods. Of these, 1.2% mentioned one correct example, 4.9% mentioned two correct examples and 5.3% mentioned three correct examples. These results are summarized in Table 2.

In the next step of analysis, the scores from the four questions were summed up to give an overall score with each question carrying maximum of 3 points. The scores ranged from 0 to 12. The nutritional knowledge levels of the mothers were generally very low. As shown on Table 2, more than half (57.4%) of the caregivers scored zero (0). Only 3.3% of the mothers had higher score of more than 9 out of 12. Applying the cut offs 0 – 5, 6 – 9 and 10–12 to group the participants as low score, medium score and high score, respectively, placed majority (91%) in the low score, 7% in the medium and only 2% in the high score group.

### Spearman's Bivariate Correlation Between Nutrition Knowledge score, Sociodemographics and Household Economics

A Spearman's correlation matrix between the mother's nutrition knowledge score, socio-demographic and household economic characteristics of the respondents is shown in the Table 5. Mothers' educational level had a modest positive significant relationship with their nutritional knowledge scores (r=0.22, *P*<0.001, α=0.01). This showed that mothers with higher educational levels are more knowledgeable in nutrition as compared with those of lower educational levels.

### Mothers' Perceptions and Attitudes Regarding Infant and Young Child Feeding

Maternal perceptions regarding infant and young child feeding was assessed regarding feeding the newborn on colostrum, initiation of breastfeeding, sufficiency of breast milk for the first 6 months and various breastfeeding and complementary feeding practices. A three-point Likert-like scale was used to assess whether they agreed, disagreed or were neutral about the assessment statements. Table 4 shows the responses of the mothers under the three categories. In most cases except one, the majority of the respondents agreed with the statements indicating an overall positive attitude. However, only 21% of mothers agreed with expressing breast milk to feed the child later, in the community. Three quarters (75%) of respondents did not agree with the statement on express breastfeeding. Similarly, more than 22% of the respondents did not believe that mothers working away from home should breastfeed their children. At the same time, almost 25% of the respondents did not believe that children 6–23 months should be fed more than four times in a day.

**TABLE 4:** Mother's Perceptions on Infant and Young Child Feeding

Statements	Agree (%)	disagree (%)	Not sure (%)
Colostrum should be given to a newborn	93.0	5.7	1.3
Early initiation of exclusive breastfeeding is beneficial to a newborn	94.9	4.6	.4
Breast milk contains sufficient water	91.7	6.1	2.2
Babies should be breastfed without being given any other food including water, up to 6 months	93.8	5.3	.8
Babies should be breastfed on demand, at all times including night time	99.6	.0	.4
Complementary foods should be introduced at 6 months	95.0	4.5	.4
After introduction of complementary foods, there should be continued breastfeeding	98.8	.8	.4
Mothers working away from home should breastfeed	75.4	22.8	1.7
A mother working away from home can express breast milk for the baby to use while she is away	21.8	75.2	2.9
Breastfed Children 6–23 months should be fed more than four times a day	73.8	24.4	1.8
Children over 6 months should be fed more than four food groups a day	87.4	9.5	3.2
Women should eat more foods during pregnancy and lactation	94.2	5.0	.8

**FIGURE 1:**
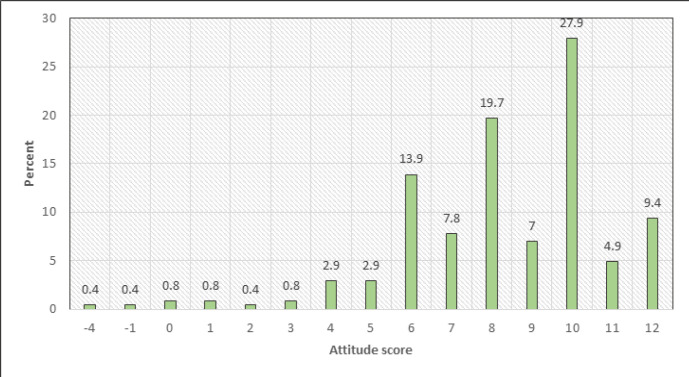
Mothers' Attitude Scores

In the next analysis step of the mothers' attitudes, responses were scored such that those who gave a positive response to the statement was awarded a score of one, those who disagreed with the statement was awarded a negative score and those who had neural score were awarded zero. The attitude score ranged from – 4 to 12 with a mean of 8.3 ± 2.6 and a median of 8. Figure 1 describes the attitude scores by mothers. Majority (90.6%) had a score of 6 or more with most (27.9%) recording a score of 10.

### Spearman Correlation Between Mothers' Attitude Score and Other Variables

Table 5 describes the Spearman's correlation between the mothers' attitude scores, socio-demographic and economic characteristics of the mothers. The respondents' attitude score demonstrated a significant positive correlation with educational level of the respondent (r=0.192, *P*<0.003, α=0.01) and maternal nutritional knowledge score (r =0.154, *P*<0.016, α = 0.05). This shows that higher educational level of the respondent is associated with better attitudes and higher nutritional knowledge.

**TABLE 5:** Spearman's Bivariate Correlation Analysis

Correlation between knowledge score and other variables			
	**Mother's Education Level**	**Mother's Age**	**Household Wealth Index**
Correlation coefficient	220**	0.024	−0.015
*P* Value	0.001	0.707	0.822
n	244	242	244
	**Mother's Education Level**	**Main occupation**	**Nutritional knowledge**
Correlation coefficient	0.192**	−0.083	0.154*
*P* Value	0.003	0.016	0.197
n	244	244	244

* Correlation is significant at the 0.05 level (2-tailed).

** Correlation is significant at the 0.01 level (2-tailed).

### Bivariate Regression Analysis of Factors Associated with Feeding of Diverse Diets

A bivariate regression analysis to determine factors associated with children's dietary diversity score showed significant positive correlation with maternal educational level (r=0.186, *P*<.000, α=.01), household wealth index (r=0.163, *P*<.000, α=.01) and the child's age (r=0.396, *P*<.004, α=.01) but not with the maternal nutritional knowledge (r=0.024, *P*>.05) and attitude score (r=0.098, *P*>.05).

## DISCUSSION

### Socio-demographics and Household Economic Characteristics

All respondents were females, majority being biological mothers to the index children. This was expected as most of the caregivers are often women. Usually, mothers or some other women in the household are charged with the role of child caring. With respect to the age of the mothers, the study found that majority were young mothers aged 35 years or less. Other studies have reported high proportions of even younger mothers in the study area. An earlier study in Kwale County for instance, reported more than 20% of the mothers were teenagers.^17^

The study also established that close to 30% of the mothers did not have any formal education while more than two-thirds had dropped out of school after class eight or did not get to finish the primary level of education. Very few mothers, (6.5%), had attained secondary school education or beyond.^17^ Low educational levels of women in Kinango have been reported in other studies.^18^ Education is one of the most important resources that enable women to provide appropriate care for their children, which is an important determinant of children's growth and development.^19^ Generally, there is a consensus that formal education is related with better incomes and employment status both of which are associated with diets and nutrition of children. Higher educational attainment among women has been strongly associated with positive nutritional outcomes in children.^20,21^

On employment status, only 1.2% of the population was in formal employment while more than half reported being housewives. As identified above, this high unemployment is highly likely a result of low educational attainment by the mothers.

The study established a positive correlation between age of the child and the dietary practices of the child. This is expected since as children grow, they get integrated into the household diets thus increasing their diversity. The result is also consistent with other studies in other parts of Kenya.^22^ In the same manner, as was alluded to in the former sections above, educational level was expected to positively influence dietary diversity in children. First educated mothers have increased chances of getting employment which means higher incomes to purchase the foods that are not produced.^23^ Secondly, educated caregivers are likely to be more conscious about the health of their children in addition to having high capacity to understand the need for feeding their children in diverse diet compared to the not educated women.

The household economic status parameterized by the Principle Component Analysis (PCA) method using house ownership properties and characteristics of the household dwelling and access to water and sanitation facilities classified one third of the households as poor. A look at the household expenditure pattern found that only 25% of the households spent more than Ksh. 10,000 in a month. This can be linked to the community depending on farming on one side or the high unemployment level in the area linked to low educational levels, a classical risk factor for poor dietary and nutritional outcomes.

The low level of education and economic status in Matuga and Kinango sub-counties of Kwale reveal the need for: Firstly, nutrition education of caregivers and other household members' in-order to ensure high nutrition knowledge levels, positive attitudes and adoption of healthy eating practices and secondly, interventions to diversify income-generating opportunities through for example kitchen gardening to promote year-round production of nutritious foods including fruits and vegetables.^26^

### Mothers' Knowledge in Nutrition

While food choices and eating behavior is a complex phenomenon influenced by many varied factors, nutrition knowledge plays a key role. The study assessed the mother's knowledge of food groups and further assessed their knowledge of the food's rich in energy, body building foods and protective foods. The study however, revealed lack of accurate nutrition knowledge among the mothers in this community. More than half of them scored zero (0) with very few scoring high in the assessment. Inadequate nutritional knowledge of caregivers has been reported by other studies in Kwale County.^17^ This can relate to the low educational level of the mothers which makes it difficult to retain the knowledge. This requires constant nutrition education through many avenues including use of mass media campaigns to ensure retention of the information. Another possible cause of this inaccurate information maybe from the neighbors, family members and friends which also forms a substantial source of the information in the community as was reported.^17^ This highlights important knowledge gaps in the study area and an urgent need to implement interventions that includes nutrition education to sensitize mothers about dietary diversity.

In a bivariate regression analysis, a positive significant correlation between mother's educational level and nutritional knowledge indicated the higher educational level was associated with higher nutritional knowledge. This is similar to what was found in another study, where it was shown that mother's nutritional knowledge increases parallel with education level.^22^

### Mothers' Attitudes and Perceptions on Infant and Young Child Feeding

The study also assessed the mothers' attitudes and perceptions regarding various child feeding recommendations including feeding the newborn on colostrum, initiation of breastfeeding, sufficiency of breast milk for the first 6 months and various breastfeeding and complementary feeding practices. Overall, the attitude of the caregivers was positive revealing willingness to practice the recommendations. However, more effort will have to be put in to promote acceptance of express breastfeeding in the study area. Most mothers did not welcome the practice, perhaps because it is a new practice that others may consider not part of their culture. Other studies have also reported similar results about express breastfeeding.

One such study was conducted in Uganda and it aimed at exploring working mothers' knowledge, perceptions and breast milk expression practices. The study reported positive association between perception of expressing breast milk and educational level.^24^ Similarly, a study in Kenya reported a positive correlation between maternal nutritional knowledge and attitude score.^22^

The lack of association between children's dietary diversity and maternal nutritional knowledge and attitudes was however contrary to expectations. Higher nutrition knowledge of caregivers has been associated with healthy dietary habits in the children in other studies. For example, two separate studies in western Kenya showed attitude was not directly associated with improvement in child's dietary diversity and suggested that it may however play an indirect role when there is adequate knowledge.^22,25^ This means that good knowledge was necessary in addition to appropriate attitudes. An intervention study in Kenya that used modeling suggested that improved nutrition knowledge was necessary in mediating the impact of seasonal variations in dietary intakes occasioned by fluctuating food production among smallholder farmers.^22^

## CONCLUSION

The findings of this study establish factors such as mothers' level of education, household wealth index and child's age to be associated with consumption of diverse diets by children aged 6–23 months. Mothers who had attained higher level of education or lived in wealthy households had children with high dietary diversity scores. In the study area however, mothers had low levels of education and a few households were ranked wealthy thus a low percentage of only 8.2% of children met the minimum dietary diversity score. There is need for targeted nutrition education and implementation of interventions to better livelihoods of the community.

## References

[1] Clark H, Coll-Seck AM, Banerjee A, et al. A future for the world's children? A WHO–UNICEF–Lancet Commission. Lancet. 2020;395(10224):605–658. 10.1016/S0140-6736(19)32540-1 32085821

[2] UNICEF, WHO and World Bank Group. Levels and Trends in Child malnutrition, 2020 Edition. 2020 Ed. Published online 2020: 1–15. https://www.unicef.org/reports/joint-child-malnutrition-estimates-levels-and-trends-child-malnutrition-2020

[3] Black RE, Morris SS, Bryce J. Where and why are 10 million children dying every year? Lancet. 2003;361(9376):2226–2234. 12842379 10.1016/S0140-6736(03)13779-8

[4] WHO. Global Strategy for Infant and Young Child Feeding. Fifthy-fourth world Heal Assem. 2003;(1):8.

[5] Molani-Gol R., Kheirouri S. & Alizadeh M. Does the high dietary diversity score predict dietary micronutrients adequacy in children under 5 years old? A systematic review. J Health Popul Nutr 42, 2 (2023). 10.1186/s41043-022-00337-3 36609429 PMC9817313

[6] World Bank. Repositioning Nutrition as Central to Development: A Strategy for Large-Scale Action. Vol 13.; 2006.

[7] Victora CG, De Onis M, Hallal PC, Blössner M, Shrimpton R. Worldwide timing of growth faltering: revisiting implications for interventions. Pediatrics. 2010;125(3):473–480. 10.1542/peds.2009-1519 20156903

[8] MOH, GOK, WHO & UNICEF National Maternal, Infant and Young Child Nutrition.; 2013.

[9] Brenda Ahoya, Justine A. Kavle, Sarah Straubinger, Constance M. Gathi, Accelerating progress for complementary feeding in Kenya: Key government actions and the way forward, Maternal & Child Nutrition, 10.1111/mcn.12723, 15, S1, 2019. PMC6594063 30748122

[10] GOK. National Food Security and Nutrition Policy.; 2007.

[11] KDHS. Kenya Demographic and Health Survey 2014. Published online 2014.

[12] Kothari M, Noureddine A. Nutrition Update 2010. 2010;(September).

[13] Desalegn BB, Lambert C, Riedel S, Negese T, Biesalski HK. Feeding Practices and Undernutrition in 6–23-Month-Old Children of Orthodox Christian Mothers in Rural Tigray, Ethiopia: Longitudinal Study. Nutrients. 2019; 11(1):138. 10.3390/nu11010138 30634659 PMC6356195

[14] Issaka AI, Agho KE, N. Page A, L. Burns P, Stevens GJ, Dibley MJ. The problem of suboptimal complementary feeding practices in West Africa: What is the way forward? Matern Child Nutr. 2015;11(May):53–60. doi:10.1111/mcn.12195 26364791 PMC6860178

[15] Kaneko S, K'opiyo J, Kiche I, et al. Health and demographic surveillance system in the western and coastal areas of kenya: An infrastructure for epidemiologic studies in Africa. J Epidemiol. 2012;22(3):276–285. doi:10.2188/jea.JE20110078 22374366 PMC3798630

[16] Fisher A, Laing J, Stockel J, Townend J. Handbook for Family Planning Operations Research Design.; 1998.

[17] Wekesa NM, Makokha A, Wanjihia VW., Lihana R, Kaneko S, Karama M. Infant feeding knowledge and practices among lactating mothers in Kwale County, Kenya. East Afr Med J. 2017;94(10):855–862.

[18] Chimerah R, Shauri H, Wokabi F, Dzoga M. Determinants of Women Participation in Livelihood Development Activities: A Case of Kinango Sub-County, Kwale County, Kenya. Int J Humanit Soc Sci. 2018;8(8):57–64. doi:10.30845/ijhss.v8n8p6

[19] Prickett KC, Augustine JM. Maternal Education and Investments in Children's Health. J Marriage Fam. 2016;78(1):7–25. doi:10.1111/jomf.12253 26778853 PMC4712746

[20] Ressa Andriyanu Utami, Agus Setiawan, Poppy Fitriyani, Identifying causal risk factors for stunting in children under five years of age in South Jakarta, Indonesia, Enfermería Clínica, Volume 29, Supplement 2, 2019, Pages 606–611, ISSN 1130-8621.

[21] Smith LC, Haddad L. Explaining Child Malnutrition in Developing Countries. Lawrence Haddad; 2000.

[22] Oduor FO, Boedecker J, Kennedy G, Mituki-Mungiria D, Termote C. Caregivers' nutritional knowledge and attitudes mediate seasonal shifts in children's diets. Matern Child Nutr. 2019;15(1):1–10. doi:10.1111/mcn.12633 PMC6859406 29968334

[23] Mayén AL, Marques-Vidal P, Paccaud F, Bovet P, Stringhini S. Socioeconomic determinants of dietary patterns in low- and middle-income countries: A systematic review. Am J Clin Nutr. 2014;100(6):1520–1531. doi:10.3945/ajcn.114.089029 25411287

[24] Okonya JN, Nabimba R, Richard M, Ombeva EA. Perceptions of breast milk expression practices among working mothers. African J Midwifery Women's Heal. 2017;11(4):169–175. doi:10.12968/ajmw.2017.11.4.169

[25] Waswa LM, Jordan I, Herrmann J, Krawinkel MB, Keding GB. Community-based educational intervention improved the diversity of complementary diets in western Kenya: Results from a randomized controlled trial. Public Health Nutr. 2015;18(18):3406–3419. doi:10.1017/S1368980015000920 25857703 PMC10271391

[26] Galhena DH, Freed R, Maredia KM. Home gardens: A promising approach to enhance household food security and wellbeing. Agric Food Secur. 2013;2(1):1–13. doi:10.1186/2048-7010-2-8

